# Deprescription in Palliative Care

**DOI:** 10.7759/cureus.39578

**Published:** 2023-05-27

**Authors:** Joana A Cabrera, Margarida Mota, Carmen Pais, Anabela Morais

**Affiliations:** 1 Internal Medicine, Centro Hospitalar Vila Nova de Gaia/Espinho, Vila Nova de Gaia, PRT; 2 Infectious Diseases, Centro Hospitalar Vila Nova de Gaia/Espinho, Vila Nova de Gaia, PRT; 3 Internal Medicine, Centro Hospitalar de Entre Douro e Vouga, Santa Maria da Feira, PRT; 4 Internal Medicine, Centro Hospitalar de Trás os Montes e Alto Douro, Vila Real, PRT

**Keywords:** goals of care, disease trajectory, polypharmacy, limited life expectancy, psychosocial effects, deprescribing criteria, preventive drugs, deprescription, palliative care

## Abstract

Individuals with limited life expectancy represent a significant proportion of healthcare consumers and are usually patients with multiple diseases and high levels of frailty. Polypharmacy and the prescription of long lists of drugs are frequent in patients with reduced life expectancy and often, as the patient’s health status deteriorates, the list of drugs increases substantially as new medications are introduced to address new symptoms or complications.

A key priority for healthcare professionals managing the care of these patients should be balancing the pharmacological approach to chronic diseases with the palliation of acute symptoms and complications. An important element of this process is to ensure that the benefit of any prescription decision outweighs potential risks. We reviewed the pros and cons of deprescribing drugs in individuals with limited life expectancy, how to identify the expected disease trajectory, which drugs are to be discontinued, identified some models trying to achieve rigorous deprescribing criteria, and the psychosocial effects of deprescribing in late phases of life.

Deprescribing is not a one-time event but rather a continuous process that requires ongoing evaluation and monitoring. It is vital to continuously monitor and evaluate the pharmacological and non-pharmacological prescriptions for patients with chronic illnesses to align them with their goals of care and life expectancy.

## Introduction and background

Individuals with limited life expectancy (less than one year) represent a significant proportion of healthcare consumers and, due to their characteristics, are at a higher risk of secondary effects of polypharmacy. Polypharmacy is defined as the regular administration of more than five drugs simultaneously and, in susceptible individuals, can contribute to a higher percentage of adverse drug effects [1-5].

As individuals age and their diseases progress, there are changes in the pharmacodynamics and pharmacokinetics of drugs. This scenario increases the likelihood of experiencing harmful drug reactions and may potentially diminish their benefit. Thus, with advancing age, polypharmacy is associated with a higher risk of adverse events secondary to medications due to drug-drug and drug-disease interactions [3-5].

It is also important to note that this population presents a dynamic health state, with often rapid and drastic changes; therefore, the use of medication in this age group should be regularly re-evaluated and adjusted [1].

A significant portion of people nearing the end of their life still consume preventative medications for chronic illnesses, as well as other medications that may be unsuitable or unnecessary. However, with disease progression and subsequent patient fragility, the change from curative to palliative therapy, with a focus on patient comfort, should translate into changes in prescription [1,3,4,6,7].

In the past years, the term “deprescription,” a way of rationalizing medication that provides limited benefit to patients, has gained prominence and has been increasingly discussed with the advent of palliative care [8,9].

## Review

Therapeutic changes at the end of life

The population with limited life expectancy presents particularities that must be considered when prescribing a therapeutic regimen. Most of these patients are elderly and have multiple comorbidities, as well as other geriatric conditions such as dementia and functional impairment, situations that condition a high degree of frailty.

Polypharmacy and the prescription of long lists of drugs are common in patients with reduced life expectancy and, often, as the patient’s health status deteriorates, the list of drugs increases substantially as new medications are introduced to address new symptoms or complications [3]. The practice of “cascade prescribing,” where a medication is prescribed to manage the adverse effects of another medication, also contributes to the build-up of prescribed pills and creates challenges in discontinuing other specific drugs.

Apart from the heightened possibility of polypharmacy and consequent adverse outcomes, administering preventative drugs to individuals with decreased life expectancy can have significant psychological implications and financial implications (patients not covered by health insurance see pharmaceutical expenses as a heavy financial burden) [1].

A key priority for healthcare professionals managing the care of these patients should be to balance the pharmacological approach to long-term diseases with the palliation of immediate symptoms and complications. An essential aspect of this procedure is to guarantee that the advantages of any prescription choice surpass the potential risks [1,10]. The ideal prescription should still be based on efficacy (including contemplation of the number of medications required to address the most extensive array of illnesses and symptoms) and safety (including weighing at what point the use of multiple medications becomes likely to cause burden), as well as the appropriateness of the prescription and its cost-effectiveness [3]. These considerations are crucial when reviewing a patient’s chronic/long-term therapy.

Primary prophylaxis or preventive therapy is defined as medications employed preemptively to manage a disease or symptom, even before the exacerbation of that condition. Usually, people in the general population considered “healthy” are medicated with long-term effect therapies, such as anti-dyslipidemics, particularly statins, antihypertensives, or bisphosphonates, among a long list of very common drugs. However, when a patient is diagnosed with a terminal illness, the benefit of these preventive or prophylactic long-term measures often becomes insignificant, and potential side effects may outweigh the benefits [1].

The time to benefit of drugs in primary prophylaxis, the time period required for a drug to demonstrate its value in a person’s health status, should be considered, as it can extend beyond the life expectancy of an individual in a palliative care context. Therefore, there is a need to evaluate prescriptions for individuals with a reduced life expectancy and consider discontinuing medications that do not unequivocally benefit the expected survival time and its quality for a particular patient. The safety, efficacy, and benefits of statins, antihypertensives, and oral antidiabetics (among other pharmacological classes) have been demonstrated for the general population through various studies, but the evidence is limited in the case of populations with reduced life expectancy, in part because this population group is rarely included in clinical trials [1]. Despite the abundance of literature analyzing potentially unsuitable medication utilization in older individuals, limited research is dedicated to preventive drugs in individuals with decreased life expectancy. However, it is always important to consider that these medications are often associated with the control/stabilization of the disease and its symptoms - the possibility of symptomatic exacerbations of the disease should always be considered when discontinuing medications [8].

The findings in the consulted literature highlight agents for lowering blood lipid levels as the most frequently identified futile or inadequate drugs in end-of-life prescriptions. Among this class of pharmacological agents, statins must be highlighted as they are the most commonly prescribed drug [5,11]. Statins are prescribed for cardiovascular and cerebrovascular diseases as primary prevention, before the first manifestation of the disease, as well as secondary prevention, to minimize the effects of a disease that is already present to prevent its recurrence. It is estimated that this class of drugs requires at least two years to present benefits to the health of users. If the primary aim of treatment is palliative care, prolonged disease prevention may be superfluous and could contribute to polypharmacy, as well as presenting an added expense for both the individual and the healthcare system [1,10]. Additionally, people who suffer from advanced disease generally have a natural decrease in circulating lipid levels due to habitual anorexia, decreased food intake, and consequent weight loss [1,12]. Finally, in addition to the well-known but slow and gradual anti-dyslipidemic effects, studies have shown an increased risk of adverse effects of statins (especially myalgias, increased serum creatine kinase, decreased muscle strength, and exercise tolerance) among people with reduced life expectancy [10].

Conducting comprehensive assessments early and frequently is crucial to detect functional changes as soon as feasible to allow informed discussions with the individual and their families to address the preferences, values, and goals of the patient, which can help guide medical care. These decisions should be made in the early and stable stages of the disease, and not in acute contexts, such as hospitalization or emergency admission, where often the patient may not have the capacity to make certain decisions [13,14].

The psychological aspect

The individual preferences of the patient are critical factors in deciding whether a medication should be prescribed and whether the patient will comply with it. Patients frequently pursue a variety of treatments independently that may not be beneficial but are also not detrimental (such as some vitamin or mineral supplements), and these can yield other advantageous outcomes, such as improved psychological well-being. Near the end of life, medications for chronic conditions that patients have been taking for several years can represent ways for patients to have some control over the end-of-life situation [7].

There are various obstacles that can impede attempts to cease the use of medications. The action of prescribing or renewing a prescription establishes a relationship between the healthcare provider and the patient, and it may be difficult for the provider to initiate discussions about discontinuing medications. Additionally, patients may resist stopping medications that they believe are necessary or that they have been taking for a long time, especially when the deprescribing process is not associated with good health education [7].

The process of deprescribing involves careful evaluation and consideration of the risks and benefits of each medication in the context of the individual patient’s health status and goals of care. Several criteria have been proposed to guide deprescribing decisions, including the patient’s life expectancy, functional status, symptom burden, comorbidities, and medication burden [1,4]. These criteria can help healthcare providers determine which medications may no longer be necessary or may be causing more harm than benefit and can assist in creating a deprescribing plan that is individualized to each patient’s unique needs and preferences.

It is important to note that deprescribing is not a one-time event, but rather a continuous process that requires ongoing evaluation and monitoring. Regular medication reviews and reassessment of the patient’s goals and preferences can help ensure that deprescribing decisions are appropriate and aligned with the patient’s wishes. Additionally, clear communication and collaboration between the healthcare team, the patient, and their caregivers is crucial to the success of deprescribing efforts and to maintaining trust and confidence in the care being provided [6].

Finally, it is important to consider the psychological impact of deprescribing on both the patient and their long-time healthcare provider. Studies suggest that clinicians may have greater difficulty discontinuing preventive medications than medications used for acute symptoms or conditions and that patients may experience feelings of abandonment or depression when medications are discontinued, particularly if they perceive this as a reduction in the quality of care being provided [8]. Thus, it is important to approach deprescribing with sensitivity and compassion, providing clear explanations of the risks and benefits of each medication and considering the patient’s emotional state and preferences at each step of the process.

Deprescription criteria

Individuals nearing the end of their life are not exclusively elderly and present diverse and fluctuating health conditions that are challenging to classify using a single measure of medication suitability [1,4].

Multiple criteria, standards, indices, and indicators have been created to categorize drug categories/appropriate medications versus unsuitable medications for the elderly or individuals with limited life expectancy, including the Beers Criteria (or Beers List), which aim to recognize medications that are deemed unsuitable for older adults and should not be initiated or should be discontinued due to an unfavorable balance between risks and benefits; the Medication Appropriateness Index, which emphasizes appropriate prescribing indices (such as indication, effectiveness, dosage, and interactions) that should be particularly considered in older individuals; and the Screening Tool of Older Persons’ Potentially Inappropriate Prescriptions (STOPP START criteria) [1,4].

In recent years, multiple studies have resulted from the study of the applicability of these criteria in the end-of-life population, resulting in new guidelines, including the prescription model developed by Holmes et al., which frames the expected life span of individuals, specific disease conditions of the patient, and the goals of therapy and the time needed to obtain benefits associated with drugs [1,3].

A diagnosis that represents reduced life expectancy redirects the goals of healthcare into a more palliative and supportive care trajectory. This change often leads to the fact that with the progression of the disease, some drugs become inappropriate.

The literature examining the appropriate use of drugs whose role is long-term primary prophylaxis among people with reduced life expectancy contains several gaps - there is no consensus on the best way to assess the relevance of drug use in the last years of expected life and various definitions of unsuitable or futile drugs are in circulation [1]. For all pharmacological classes, there are specific guidelines on when to start a certain drug, but there are rarely guidance norms on the timing of discontinuation of a certain pharmacological class [8,12]. Multiple reviews also highlight the need for standardized norms for the approach to certain pathologies in individuals with reduced life expectancy [3,13].

Definition of disease trajectories

In the approach to palliative care, there are some general indicators of bad prognosis described in the Gold Standards Framework that the clinician should identify as early as possible because they can modify the approach to a particular patient. [14] These include: (1) Serum hypoalbuminemia <2.5 g/dL. (2) Sarcopenia and weight loss equal to or greater than 5% and associated frailty/cachexia. (3) Frequent hospital admissions (defined as >2/year), and increased mortality in people over 65 years old with multiple comorbidities. (4) Sentinel events, such as repeated falls, frequent urgency episodes, or admission to a nursing home.

In the course of any chronic disease, but especially in the context of a terminal illness, moments between the clinician and the patient (and their family) should be privileged to make decisions in advance regarding the care to be provided during the course of the disease.

In defining this care, it is important to holistically address the patient’s well-being but also to address more specific decisions that often require the making of major and important decisions quickly, in situations where the patient is often not mentally or physically capable of making them, being an extra source of stress for the family. Among the most critical aspects to address are: (1) Escalation of care and resuscitation in case of cardiac arrest, according to the Do Not Resuscitate status decision of each patient. (2) Interruption of treatments, especially invasive ones, such as peripheral glucose assessments and interruption of injectable drugs. (3) Necessity and relevance of enteral feeding (as well as feeding devices such as a nasogastric tube or percutaneous endoscopic gastrostomy) or even parenteral feeding. (4) Privilege of pain control. (5) Preference for the place of care or the place for the last days/hours; as an example, most people wish to die at home and avoid long hospital stays, but less than 10% actually die in their chosen place [14].

## Conclusions

Regular monitoring and evaluation of medication therapy for patients with chronic illnesses are important. However, when a diagnosis of a life-limiting condition is made, it is vital to match it with the objectives of care and anticipated. As death approaches, changes in care goals should be continuously and regularly evaluated, and appropriate changes to medication therapy should be made when relevant.

All healthcare professionals, especially those who deal with palliative care populations, must promote optimal medication use in people with reduced life expectancy by regularly and critically evaluating their patients’ therapeutic regimens, with a special focus on considering the relevance of the continued use of preventive or pointless medications.

Any decision about maintaining or discontinuing a drug in a patient at the end of life should not only consider medical factors but also considerations about each individual’s frailty, mental and psychological state, and social situation.

There is significantly more scientific literature on when to initiate medications than when to discontinue them. Nonetheless, prioritizing and terminating medications can curtail expenses, simplify prescription schedules, lower the chances of adverse drug events and polypharmacy, focus therapies where they are most effective, and prevent the underutilization of other medications due to economic reasons.

In an area where the need for more research is consensual, any decision should be patient-centered and made in conjunction with the patient or, alternatively, with their caregivers. Moreover, the risks and benefits should be explained and weighed, and the patient’s decision should always be valued.

## References

[1] Maddison AR, Fisher J, Johnston G (2011). Preventive medication use among persons with limited life expectancy. Prog Palliat Care.

[2] Hedman C, Frisk G, Björkhem-Bergman L (2022). Deprescribing in palliative cancer care. Life (Basel).

[3] Holmes HM (2009). Rational prescribing for patients with a reduced life expectancy. Clin Pharmacol Ther.

[4] LeBlanc TW, McNeil MJ, Kamal AH, Currow DC, Abernethy AP (2015). Polypharmacy in patients with advanced cancer and the role of medication discontinuation. Lancet Oncol.

[5] Poudel A, Yates P, Rowett D, Nissen LM (2017). Use of preventive medication in patients with limited life expectancy: a systematic review. J Pain Symptom Manage.

[6] Alexander GC, Sayla MA, Holmes HM, Sachs GA (2006). Prioritizing and stopping prescription medicines. CMAJ.

[7] Rossi MI, Young A, Maher R (2007). Polypharmacy and health beliefs in older outpatients. Am J Geriatr Pharmacother.

[8] Todd A, Husband A, Andrew I, Pearson SA, Lindsey L, Holmes H (2017). Inappropriate prescribing of preventative medication in patients with life-limiting illness: a systematic review. BMJ Support Palliat Care.

[9] Todd A, Holmes HM (2015). Recommendations to support deprescribing medications late in life. Int J Clin Pharm.

[10] Kutner JS, Blatchford PJ, Taylor DH Jr (2015). Safety and benefit of discontinuing statin therapy in the setting of advanced, life-limiting illness: a randomized clinical trial. JAMA Intern Med.

[11] McGrath L, Goodlin SJ (2017). Palliative care in older adults with cardiovascular disease: addressing misconceptions to advance care. Curr Cardiovasc Risk Rep.

[12] Vandenhaute V (2010). Palliative care and type II diabetes: a need for new guidelines?. Am J Hosp Palliat Care.

[13] Sullivan MF, Kirkpatrick JN (2020). Palliative cardiovascular care: the right patient at the right time. Clin Cardiol.

[14] Dunning T, Martin P (2018). Palliative and end of life care of people with diabetes: issues, challenges and strategies. Diabetes Res Clin Pract.

